# Hepatic Phospholipid Remodeling Modulates Insulin Sensitivity and Systemic Metabolism

**DOI:** 10.1002/advs.202300416

**Published:** 2023-04-23

**Authors:** Ye Tian, Kritika Mehta, Matthew J. Jellinek, Hao Sun, Wei Lu, Ruicheng Shi, Kevin Ingram, Randall H. Friedline, Jason K. Kim, Jongsook Kim Kemper, David A. Ford, Kai Zhang, Bo Wang

**Affiliations:** ^1^ Department of Comparative Biosciences College of Veterinary Medicine University of Illinois at Urbana‐Champaign Urbana IL 61802 USA; ^2^ Department of Biochemistry School of Molecular and Cellular Biology University of Illinois at Urbana‐Champaign Urbana IL 61801 USA; ^3^ Department of Biochemistry and Molecular Biology and Center for Cardiovascular Research Saint Louis University St. Louis MO 63104 USA; ^4^ Department of Molecular and Integrative Physiology School of Molecular and Cellular Biology University of Illinois at Urbana‐Champaign Urbana IL 61801 USA; ^5^ Program in Molecular Medicine and Division of Endocrinology Metabolism and Diabetes Department of Medicine University of Massachusetts Medical School Worcester MA 01655 USA; ^6^ Cancer Center at Illinois University of Illinois at Urbana‐Champaign Urbana IL 61801 USA; ^7^ Division of Nutritional Sciences College of Agricultural Consumer and Environmental Sciences University of Illinois at Urbana‐Champaign Urbana IL 61801 USA

**Keywords:** FGF21, insulin signaling transduction, LPCAT3, phospholipid remodeling, selective insulin resistance

## Abstract

The liver plays a central role in regulating glucose and lipid metabolism. Aberrant insulin action in the liver is a major driver of selective insulin resistance, in which insulin fails to suppress glucose production but continues to activate lipogenesis in the liver, resulting in hyperglycemia and hypertriglyceridemia. The underlying mechanisms of selective insulin resistance are not fully understood. Here It is shown that hepatic membrane phospholipid composition controlled by lysophosphatidylcholine acyltransferase 3 (LPCAT3) regulates insulin signaling and systemic glucose and lipid metabolism. Hyperinsulinemia induced by high‐fat diet (HFD) feeding augments hepatic *Lpcat3* expression and membrane unsaturation. Loss of *Lpcat3* in the liver improves insulin resistance and blunts lipogenesis in both HFD‐fed and genetic *ob/ob* mouse models. Mechanistically, *Lpcat3* deficiency directly facilitates insulin receptor endocytosis, signal transduction, and hepatic glucose production suppression and indirectly enhances fibroblast growth factor 21 (FGF21) secretion, energy expenditure, and glucose uptake in adipose tissue. These findings identify hepatic LPCAT3 and membrane phospholipid composition as a novel regulator of insulin sensitivity and provide insights into the pathogenesis of selective insulin resistance.

## Graphical abstract

Hepatic phospholipid (PL) saturation, mediated by depletion of a PL remodeling enzyme LPCAT3, protects mice from diet‐induced obesity through FGF21 and elevated energy expenditure. Ablation of *Lpcat3* in the liver improves insulin sensitivity by facilitating insulin receptor endocytosis into early endosomes and glucose uptake in brown adipose tissue.

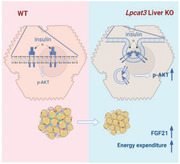

## 1 Introduction

The prevalence of type 2 diabetes (T2D) has increased dramatically due to the global rise in obesity, sedentary lifestyle, and energy‐dense diets.^[^ ^1^ ^]^ T2D is characterized by relative insulin deficiency caused by pancreas *β*‐cell dysfunction and insulin resistance (IR) in metabolic organs, including liver, muscle, and adipose tissue.^[^ ^2^ ^]^ The liver plays a central role in regulating glucose and lipid homeostasis. In normal liver, insulin binds to the insulin receptor (INSR), recruits insulin receptor substrates, and activates PI3K/Akt and its downstream effectors to stimulate glycogen synthesis and suppress gluconeogenesis.^[^ ^3^ ^]^ Additionally, insulin is known to increase de novo lipogenesis and promote the esterification and secretion of triglyceride (TG) in the liver, thereby contributing to fatty liver and hypertriglyceridemia in T2D.^[^ ^4^ ^]^ Aberrant insulin action in the liver is believed to be a major driver of insulin resistance, in which insulin fails to adequately suppress hepatic glucose production (HGP), but paradoxically continues to promote lipid biosynthesis, leading to hyperglycemia and hypertriglyceridemia.^[^ ^5^ ^]^ Therefore, the lipid‐promoting effect of insulin is maintained, while the control of HGP is defective during insulin resistance, a phenomenon known as selective insulin resistance. However, despite the extensive characterization of downstream signaling cascades in insulin's control of glucose and lipid metabolism, how insulin differentially regulates glucose and lipid metabolism in insulin resistant liver remains obscure.

Phospholipids (PLs) are major components of biological membranes that separate cellular contents from the surrounding environment, form organelles, and provide platforms for cellular processes, such as signal transduction, vesicle trafficking, and molecular transport.^[^ ^6^ ^]^ The chain length and number of double bonds in the fatty acids of PLs determine the biophysical properties of membranes, thereby affecting the function of membrane‐bound proteins and processes associated with membranes.^[^ ^7^ ^]^ In mammalian cells, PL composition is largely controlled by Lands’ cycle, in which phospholipase A2 hydrolyzes sn‐2 fatty acyl chain of PLs and lysophospholipid acyltransferase synthesizes a new PL species by incorporating another fatty acyl chain to the sn‐2 site.^[^ ^8^ ^]^ We previously identified a PL remodeling enzyme, lysophosphatidylcholine acyltransferase 3 (Lpcat3), as a major determinant of membrane PL composition in the liver.^[^ ^9^ ^]^ Lpcat3 preferentially drives the incorporation of polyunsaturated fatty acids (PUFAs) into phosphatidylcholine (PC) to generate polyunsaturated PCs. We have shown that loss of *Lpcat3* in the liver selectively reduces polyunsaturated PCs in membrane and consequently decreases membrane fluidity, resulting in impaired sterol regulatory element binding protein (SREBP)‐1c processing and lipogenesis, and very low‐density lipoprotein (VLDL) secretion.^[^ ^10^ ^]^

Here we show that high‐fat diet (HFD) feeding augments *Lpcat3* expression and increases polyunsaturated PL composition in the liver. Knockout of *Lpcat3* in the liver (LKO) improves insulin sensitivity and glucose tolerance in both HFD‐fed and *Leptin* deficient *ob/ob* mice. Furthermore, *Lpcat3* deficiency suppresses the expression of SREBP‐1c and lipogenic genes in HFD‐fed and *ob/ob* mouse liver. Mechanistic studies demonstrate that loss of hepatic *Lpcat3* enhances insulin sensitivity through both cell‐autonomous effects by facilitating insulin signal transduction in early endosomes, and nonautonomous effects by increasing FGF21 secretion, energy expenditure (EE), and glucose uptake in brown adipose tissue (BAT). These findings reveal an unrecognized reciprocal regulation between insulin signaling and PL remodeling and highlight a mechanism whereby hepatic membrane PL composition mediates both prolipogenesis and anti‐HGP effects of insulin in the liver, providing novel insight into the pathogenesis of selective insulin resistance.

## 2 Results

### 2.1 Lpcat3 Expression Is Induced by Insulin Signaling

**Figure 1:**
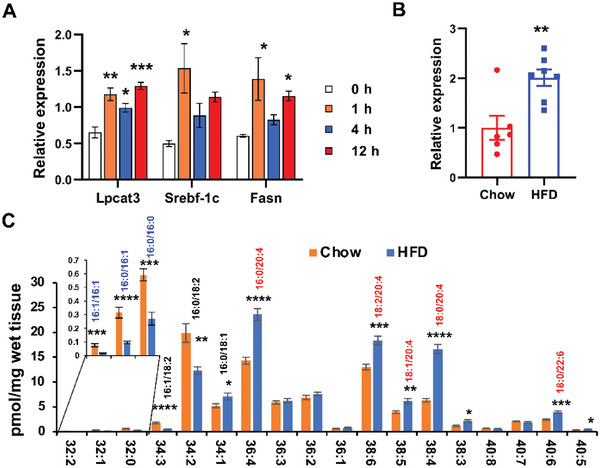
*Lpcat3* expression is regulated by insulin signaling. A) Expression of *Lpcat3* and insulin‐responsive genes (*Srebf‐1c* and *Fasn*) in Hepa 1–6 cells treated with insulin (5 nm) for different time points. B) *Lpcat3* mRNA levels in livers of *C57BL/6* mice fed chow and HFD for 16 weeks. C) Mass spectrometry analysis of phosphatidylcholine (PC) composition in livers of chow (*n* = 6) and HFD‐fed (*n* = 8) *C57BL/6* mice for 16 weeks. Data are presented as means ± SEM. Statistical analysis was performed with A) one‐way ANOVA and B,C) Student's *t*‐test. *P < 0.05, **P < 0.01, ***P < 0.001, ****P < 0.0001.

Our previous observation that *Lpcat3* expression is increased in *ob/ob* mouse liver promoted us to hypothesize that LPCAT3 and PL remodeling may be involved in the pathogenesis of insulin resistance.^[^ ^10b^ ^]^ In support of this hypothesis, insulin treatment indeed significantly increased mRNA levels of *Lpcat3* in Hepa 1–6 cells (**Figure** **1**A), which was comparable to the induction of known downstream genes of insulin, *Srebf‐1c* and *Fasn*. Furthermore, *Lpcat3* expression was increased by twofold in the livers of HFD‐fed C57BL/6 mice (Figure 1B). Mass spectrometry analysis revealed that polyunsaturated PCs, especially arachidonoyl‐containing PCs, were dramatically elevated, while saturated and monosaturated PCs were decreased in HFD‐fed livers (Figure 1C), suggesting that hyperinsulinemia in both genetic‐ and diet‐induced mouse models enhances *Lpcat3* expression and membrane PL unsaturation in the liver.

### 2.2 Loss of Lpcat3 in the Liver Enhances Insulin Sensitivity

**Figure 2:**
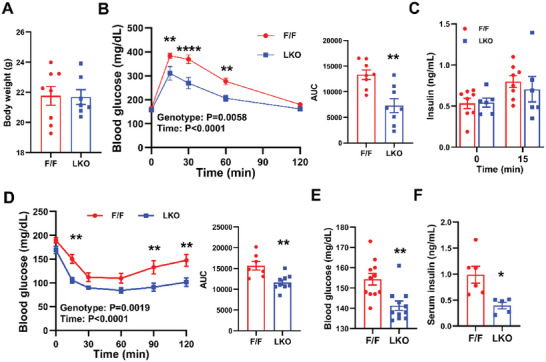
Loss of *Lpcat3* in liver enhances insulin sensitivity in chow diet‐fed mice. A) Body weight of chow diet‐fed *Lpcat3^fl/fl^* (F/F) and *Lpcat3^fl/fl^ Albumin‐Cre* (LKO) mice when Glucose tolerance test (GTT) and insulin tolerance test (ITT)were performed. B) GTT in chow diet‐fed control F/F and LKO mice. AUC: area under the curve. The *P* values of source of variation were provided. C) Serum insulin levels during GTT analysis. D) ITT in chow diet‐fed control F/F and LKO mice. E) Blood glucose and F) serum insulin levels in chow diet fed control F/F and LKO mice after fasting/refeeding. Mice were fasted overnight and refed with HFD for 2 h. Data are presented as means ± SEM. Statistical analysis was performed with Student's t test (A, AUC in B and D, E,F), and B–D) two‐way ANOVA. *P < 0.05, **P < 0.01, ****P < 0.0001.

To determine if PL composition modulates insulin sensitivity, we characterized glucose metabolism in *Lpcat3* liver‐specific knockout (LKO) mice, which exhibit opposite PL composition compared to obese and HFD‐fed livers.^[^ ^10a^ ^]^ Although the floxed control and LKO mice had similar body weight (BW) on chow diet (**Figure** **2**A), intraperitoneal glucose tolerance test showed that LKO mice cleared glucose load more efficiently than control mice (Figure 2B). Control and LKO mice had comparable serum insulin levels upon glucose injection, suggesting that enhanced glucose clearance was not caused by increased insulin secretion (Figure 2C). Insulin tolerance test (ITT) further corroborated that LKO mice were more insulin sensitive compared to control mice (Figure 2D). Insulin signaling is dynamically regulated during fasting and refeeding. To determine if LPCAT3 modulates the activity of insulin signaling during this process, we fasted LKO and control mice overnight and refed them with HFD for 2 h. Blood glucose levels were significantly lower in LKO mice, albeit much lower serum insulin levels compared to control mice (Figure 2E,F). These data demonstrated that LPCAT3 and membrane PL composition modulate insulin sensitivity under physiological conditions.

### 2.3 Lpcat3 Deficiency Protects Mice from HFD‐Induced Obesity and Insulin Resistance

**Figure 3:**
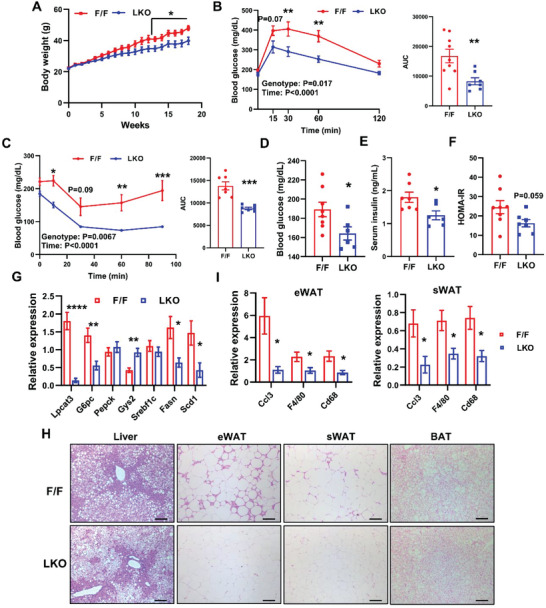
LKO mice are protected from HFD‐induced obesity and insulin resistance. A) Growth curve of control F/F and LKO mice on HFD (*n* = 7/group). B) GTT and C) ITT in HFD‐fed F/F and LKO mice. D) Fasting blood glucose, E) serum insulin levels, and F) HOMA‐IR of HFD‐fed F/F and LKO mice. G) Expression of selected genes in livers of HFD‐fed F/F and LKO mice (*n* = 6–8/group). H) Representative histology of liver, white adipose tissue (eWAT and sWAT) and brown adipose tissue (BAT) from HFD‐fed F/F and LKO mice. Scale bar: 100 µm. I) Expression of inflammation‐related genes in eWAT and sWAT of HFD‐fed F/F and LKO mice (*n* = 7–9/group). Data are presented as means ± SEM. Statistical analysis was performed with Student's t test (A, AUC in B,C, D–G, I) and B,C) two‐way ANOVA. *P < 0.05, **P < 0.01, ***P < 0.001, ****P < 0.0001.

Next, we examined if LPCAT3 and PL remodeling play any roles in HFD‐induced insulin resistance. Surprisingly, LKO mice gained significantly less body weight after 12‐week HFD feeding compared to controls (**Figure** **3**A). Magnetic resonance imaging (MRI) analysis of body composition showed that the difference in body weight was primarily due to lower whole body fat mass, particularly subcutaneous white adipose tissue (sWAT) in HFD‐fed LKO mice (Figure S1A–D, Supporting Information). Further investigation suggested that this resistance to HFD‐induced weight gain was not due to reduced food consumption or lipid absorption in LKO mice (Figure S1E–G, Supporting Information).

Similar to chow diet‐fed mice, HFD‐fed LKO mice were more glucose tolerant and insulin sensitive as demonstrated by glucose tolerance test (GTT) and ITT analyses (Figure 3B,C). Reduced fasting blood glucose, serum insulin levels, and homeostatic model assessment of insulin resistance (HOMA‐IR) further confirmed improved insulin sensitivity in LKO mice on HFD (Figure 3D–F). Gene expression analysis showed that glucose‐6‐phosphotase (*G6pc*) was downregulated, while glycogen synthase 2 (*Gys2*) was upregulated in LKO livers. The expression of lipogenic genes was significantly suppressed in the absence of *Lpcat3* (Figure 3G). LKO mice exhibited lower serum triglyceride and nonesterified fatty acid (NEFA) levels accompanied by hepatic accumulation of triglyceride and NEFA upon HFD feeding (Figure S1H–K, Supporting Information), likely due to impaired VLDL secretion.^[^ ^10a^ ^]^ Histological analysis showed much less inflammation in WAT of LKO mice compared to control mice on HFD (Figure 3H), which was supported by a significant decrease in the expression of cytokine *Ccl3* and macrophage markers *F4/80* and *Cd68* (Figure 3I). Taken together, these data indicate that *Lpcat3* deficiency improves both hyperglycemia and hypertriglyceridemia in HFD‐induced insulin resistance.

### 2.4 Loss of Lpcat3 Improves Insulin Resistance in ob/ob Mice

**Figure 4:**
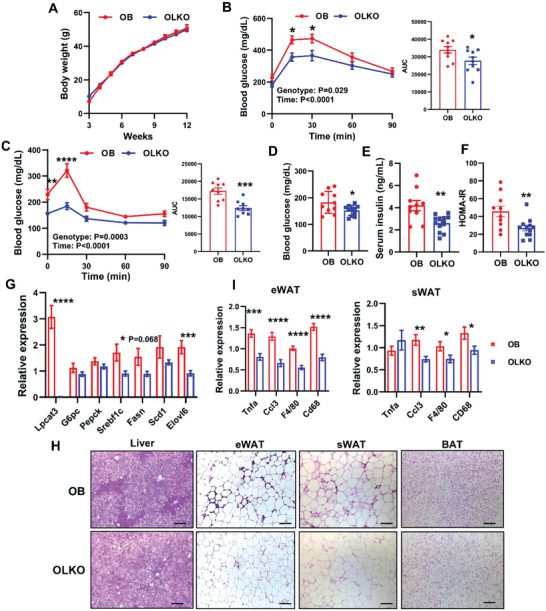
Loss of *Lpcat3* in the liver improves insulin resistance in *ob/ob* mice. A) Growth curve of *ob/ob, Lpcat3^fl/fl^* (OB), and *ob/ob, Lpcat3^fl/fl^ Albumin‐Cre* (OLKO) mice (*n* = 10/group). B,C) GTT and ITT in OB and OLKO mice. D) Fasting blood glucose, E) serum insulin levels, and F) HOMA‐IR of OB and OLKO mice. G) Expression of selected genes in the livers of OB and OLKO mice (*n* ≥ 10/group). H) Representative histology of liver, epididymal white adipose tissue (eWAT), subcutaneous white adipose tissue (sWAT), and BAT from OB and OLKO mice. Scale bar: 100 µm. I) Expression of inflammation‐related genes in eWAT and sWAT of OB and OLKO mice (*n* ≥ 10/group). Data are presented as means ± SEM. Statistical analysis was performed with Student's *t*‐test (A, AUC in B,C, D–G, I) and B,C) two‐way ANOVA. *P < 0.05, **P < 0.01, ***P < 0.001, ****P < 0.0001.

To further assess the involvement of LPCAT3 activity in the pathogenesis of insulin resistance, we crossed LKO mice with *ob/ob* mice to generate *ob/ob*, *Lpcat3* ^fl/fl^, *Cre‐* (OB) and *ob/ob*, *Lpcat3* ^fl/fl^, *Cre+* (OLKO) mice. Unlike HFD‐fed mice in C57BL/6 background, knockout of *Lpcat3* in the liver did not affect body weight gain in *ob/ob* mice (**Figure** **4**A and Figure S2A, Supporting Information). There was a slight decrease in the weight of epididymal WAT (eWAT), but not sWAT in OLKO mice (Figure S2B,C, Supporting Information). OLKO and OB mice had comparable daily food consumption (Figure S2D, Supporting Information).

Despite no body weight differences, OLKO mice were more glucose tolerant and insulin sensitive compared to OB mice (Figure 4B,C). Consistently, OLKO mice showed lower fasting blood glucose, serum insulin levels and HOMA‐IR (Figure 4D–F). The expression of *Srebp‐1c* and lipogenic genes was reduced in OLKO livers compared to that in OB livers, while the expression of gluconeogenic genes was not altered (Figure 4G). Surprisingly, OLKO mice had increased serum triglyceride and NEFA levels, whereas hepatic triglyceride and NEFA levels were comparable (Figure S2E–H, Supporting Information). Similar to HFD‐fed mice, the inflammatory cell infiltration as well as the expression of macrophage markers and cytokines in WAT were dampened in OLKO mice (Figure 3H,I). Notably, the expression of lipase genes, *Hsl* and *Atgl*, was significantly upregulated in eWAT but not in sWAT in OLKO mice (Figure S2I, Supporting Information), which may be responsible for smaller eWAT but not sWAT in OLKO mice, and likely contributes to an increase in serum NEFA levels in OLKO mice.

### 2.5 Lpcat3 Deficiency Increases Insulin Sensitivity Partially through Facilitating Insulin Signal Transduction in Early Endosomes

Several lipid classes, such as free fatty acids (FFAs), diacylglycerol (DAG), and ceramides, are known to mediate insulin resistance in the liver.^[^ ^11^ ^]^ To determine if loss of *Lpcat3* affects the levels of these lipids, we performed a shotgun lipidomic analysis in both chow and HFD‐fed mouse livers. In agreement with impaired VLDL secretion, a majority of lipid classes were increased in LKO livers compared to controls (Figure S3A,B, Supporting Information), including ceramide, FFAs, DAG, and TG (Figure S3C–F, Supporting Information). In agreement with the enzyme activity of LPCAT3 that prefers unsaturated fatty acids (FA) as substrate, unsaturated FA, but not saturated FA, was increased in LKO livers (Figure S3G, Supporting Information). Prostaglandin E2 (PGE2), a product of arachidonic acid metabolism, has been shown to contribute to insulin resistance.^[^ ^12^ ^]^ The levels of PGE2 were comparable between control and LKO mice on chow or HFD (Figure S3H, Supporting Information), suggesting that PGE2 is unlikely to be involved in the regulation of insulin signaling in the absence of *Lpcat3*.

**Figure 5:**
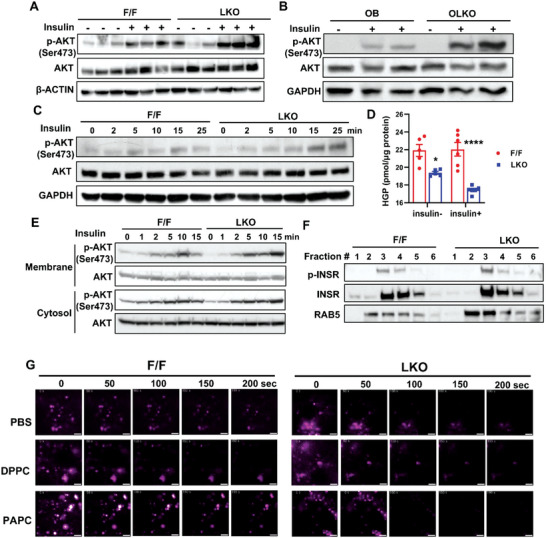
*Lpcat3* deficiency enhances insulin signal transduction in hepatocytes through facilitating insulin receptor internalization to early endosomes. A) Western blot analysis of p‐AKT (Ser473) levels in livers from chow diet‐fed F/F and LKO mice treated with insulin. Female mice were fasted overnight and i.p. injected with insulin (1 U kg^−1^ BW) for 15 min. B) Western blot analysis of p‐AKT levels in livers from OB and OLKO mice treated with insulin. Mice were fasted overnight and retro‐orbitally injected with insulin (2 U kg^−1^ BW) for 15 min. C) Western blot analysis of p‐AKT levels in primary hepatocytes treated with insulin. Primary hepatocytes were isolated from F/F and LKO mice and treated with insulin (1 nm) for different time points. D) Hepatic glucose production (HGP) assay. Primary hepatocytes were isolated from F/F and LKO mice, cultured in glucose free medium for 6 h before treated with lactate and pyruvate with or without insulin (10 nm) for 3 h. E) Western blot analysis of p‐AKT levels in membrane and cytosol fractions of purified from livers of insulin treated mice. Chow diet‐fed F/F and LKO mice were fasted overnight and injected with insulin (1 U kg^−1^ BW) through vena cava for different time points. F) Western blot analysis of p‐INSR in early endosomes isolated from livers of F/F and LKO mice treated with insulin. Mice were fasted overnight and injected with insulin (1 U kg^−1^ BW) for 5 min. G) Representative images of TIRF microscopy of primary hepatocytes isolated from F/F and LKO mice and treated with liposomes followed by Cy3‐insulin. D) Data are presented as means ± SEM. Statistical analysis was performed with two‐way ANOVA. *P < 0.05, ****P < 0.0001.

To explore the mechanisms by which PL composition modulates insulin sensitivity, we first determined if insulin signaling was altered in LKO livers. Western blot analysis showed that insulin‐stimulated expression of phospho‐AKT (p‐AKT) levels were increased in chow diet‐fed LKO mice (**Figure** **5**A). Similarly, p‐AKT levels were dramatically enhanced in OLKO livers (Figure 5B), suggesting that *Lpcat3* deficiency potentiates hepatic insulin signaling. To further test cell autonomous effects of LPCAT3 on insulin signaling and its kinetics, we treated primary hepatocytes with insulin for different time points and measured p‐AKT levels. Similar to in vivo data, p‐AKT levels were elevated in LKO hepatocytes and remained higher at 25 min post‐treatment, while p‐AKT levels started to decrease in control hepatocytes at this time (Figure 5C). This indicates that *Lpcat3* deficiency not only increases the intensity but also extends the duration of insulin signaling. Consistent with enhanced insulin sensitivity, primary hepatocytes isolated from LKO mice showed dramatic reduction in glucose production compared to controls (Figure 5D). Thus, these data support a cell‐autonomous mechanism involving alterations in PL composition.

Insulin signaling is regulated by the abundance and compartmentalization of INSR in the plasma membrane.^[^ ^13^ ^]^ Western blot analysis showed no difference in INSR protein levels between control and LKO livers (Figure S4A, Supporting Information). It has been shown that increasing membrane saturation facilitates the compartmentalization and activity of membrane‐bound proteins by recruiting them to microdomains.^[^ ^14^ ^]^ Our data showed that INSR was primarily localized in CD71 enriched nonraft domains (fraction #3 and #4) in plasma membrane (Figure S4B, Supporting Information). Furthermore, insulin treatment did not drastically alter INSR localization. These data indicate that increased insulin sensitivity in LKO mice is unlikely caused by changes in INSR expression or its distribution/recruitment to different compartments in plasma membrane.

Upon insulin binding, INSR internalizes into early endosomes, which become signaling endosomes and comprise an important compartment in insulin signal transduction.^[^ ^15^ ^]^ Given that membrane curvature, largely determined by membrane PL composition, regulates endocytosis,^[^ ^16^ ^]^ we first examined the insulin signal intensity in different subcellular compartments. As shown in Figure 5E, p‐AKT levels were higher in both plasma membrane and cytosol in LKO livers, further corroborating our observations in primary hepatocytes. Next, we purified endosomes from livers of control and LKO mice retro‐orbitally injected with insulin for 5 min. We found that RAB5, a marker of early endosomes, was enriched in fractions #2 and #3 in LKO livers compared to control livers (Figure 5F), despite comparable RAB5 levels in whole tissue lysates from control and LKO livers (Figure S4C, Supporting Information). Moreover, INSR and phosphorylated INSR levels were higher in early endosomes of LKO livers. Next, we directly visualized insulin‐dependent INSR internalization using total internal reflection (TIRF) microscopy. We monitored the kinetics of insulin endocytosis in control and LKO primary hepatocytes treated with Cy3‐insulin. We found that Cy3‐insulin signals started to decrease within 50 s and disappeared ≈100 s after treatment in LKO hepatocytes (Figure 5G and Video S1, Supporting Information). In contrast, most of Cy3‐insulin signals remained on plasma membrane during the course of recording of ≈200 s in control hepatocytes (Figure 5G and Video S2, Supporting Information). In comparison, transferrin endocytosis was not affected by *Lpcat3* deficiency (Figure S4C and Videos S3 and S4, Supporting Information). To determine if altered endocytosis is caused by changes in membrane PL composition, we treated primary hepatocytes with liposomes containing 16:0/16:0 PC (DPPC) or 16:0/20:4 PC (PAPC) and monitored Cy3‐insulin internalization. Indeed, dipalmitoylphosphatidylcholine (DPPC) treatment promoted Cy3‐insulin internalization (Figure 5G and Video S5, Supporting Information), whereas 1‐palmitoyl‐2‐arachidonoyl‐sn‐glycero‐3‐phosphorylcholine (PAPC) had little effect in control hepatocytes (Figure 5G and Video S6, Supporting Information). In contrast, the impact of liposomes on LKO hepatocytes was not as pronounced as in control hepatocytes (Figure 5G and Videos S7 and S8, Supporting Information). Mass spectrometry analysis showed that DPPC levels were increased by more than tenfold after liposome treatment, while PAPC levels were only slightly increased in control hepatocyte and failed to rise in LKO hepatocytes (Figure S4E, Supporting Information), indicating that PLs in liposomes undergo active remodeling process and PAPC cannot be incorporated into membrane in the absence of LPCAT3. Nevertheless, these data suggest that loss of *Lpcat3* likely enhances insulin sensitivity through facilitating INSR internalization and insulin signal transduction in early endosomes.

### 2.6 Loss of Lpcat3 Increases Systemic Insulin Sensitivity by Enhancing Glucose Uptake in Brown Fat

**Figure 6:**
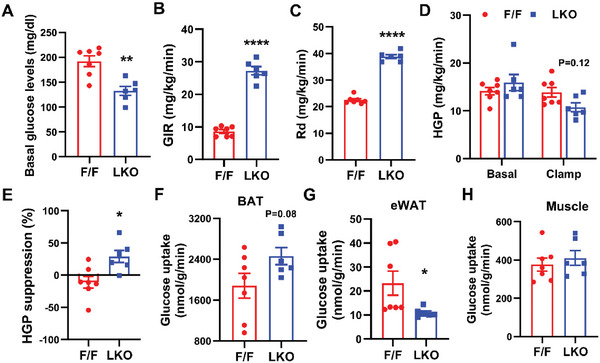
Loss of *Lpcat3* in liver suppresses HGP and increases glucose uptake in brown adipose tissue (BAT). A–H) Hyperinsulinemic‐euglycemic clamp analysis of F/F and LKO mice fed with HFD for 12 weeks. A) Basal glucose levels, B) glucose infusion rate (GIR), C) whole‐body glucose turnover (Rd), D) HGP at basal and during insulin clamp, E) insulin‐mediated suppression of HGP, and F–H) insulin‐stimulated glucose uptake in intrascapular BAT, eWAT, and gastrocnemius skeletal muscle (*n* = 6–7/group). Data are presented as means ± SEM. Statistical analysis was performed with A–C,E–H) Student's *t*‐test and D) two‐way ANOVA. *P < 0.05, **P < 0.01, ****P < 0.0001.

Our observations that LKO mice were less obese on HFD and were protected from adipose tissue inflammation led us to speculate that peripheral insulin sensitivity might be altered in these mice. To examine this further, we performed hyperinsulinemic‐euglycemic clamp experiments in awake HFD‐fed control and LKO mice. The clamp data indicate that LKO mice are more insulin sensitive than control mice, as demonstrated by a marked decrease in basal glucose levels, and more than two to threefold increases in glucose infusion rate (GIR) and whole‐body glucose turnover (Rd) in LKO mice during the clamps (**Figure** **6**A–C). While basal HGP rates were similar, the HGP during clamps was decreased by ≈20% in LKO livers compared to controls (Figure 6D). As a result, LKO mice exhibited a higher insulin‐mediated suppression of HGP (Figure 6E). Insulin‐stimulated glucose uptake in BAT was increased by ≈30%, whereas glucose uptake was not altered in muscle but reduced in eWAT in LKO mice (Figure 6F–H). However, compared to BAT and muscle, the contribution of WAT in glucose disposal was negligible. Taken together, these data suggest that both suppressed glucose production in the liver and increased glucose uptake in the BAT contribute to the improved insulin sensitivity in HFD‐fed LKO mice.

### 2.7 Lpcat3 Deficiency Induces FGF21 Secretion and Enhances Energy Expenditure

**Figure 7:**
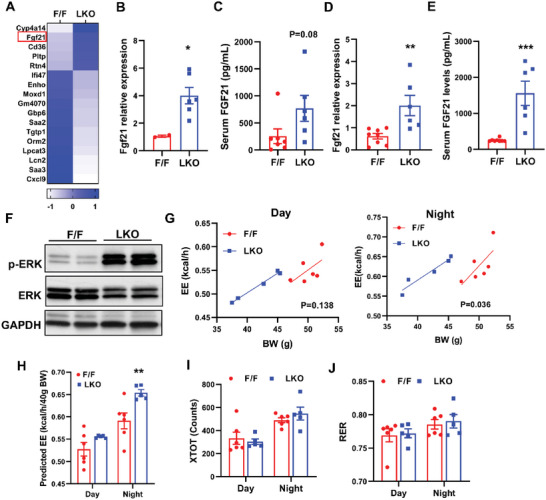
Loss of *Lpcat3* in the liver increases FGF21 secretion and energy expenditure (EE). A) Heatmap of dysregulated genes based on previously published microarray data in livers of chow diet‐fed F/F and LKO mice (*n* = 5/group). *Fgf21* mRNA levels in B) the livers and C) serum FGF21 levels of chow diet fed F/F and LKO mice. *Fgf21* mRNA levels in D) the livers and E) serum FGF21 levels (Ea) of HFD fed F/F and LKO mice. F) Western blot analysis of p‐ERK in eWAT of HFD‐fed F/F and LKO mice. G) EE analyzed by regression analysis of covariance (ANCOVA). H) EE estimated by univariate generalized linear model (GLM) with body mass set to 46.3 g (average body mass of F/F and LKO mice on HFD). I) Physical activity and J) respiratory exchange ratio (RER) in HFD‐fed F/F and LKO mice. Data are presented as means ± SEM. Statistical analysis was performed with B–E) Student's *t*‐test, G) ANCOVA, and H–J) two‐way ANOVA. *P < 0.05, **P < 0.01, ***P < 0.001.

Next, we explored the mechanisms by which changes in hepatic PL composition affect body weight gain and glucose uptake in BAT. Our previous published transcriptional profiling in chow diet‐fed mice showed that despite marked changes in membrane composition in LKO livers, the effect on gene expression was limited on chow diet.^[^ ^10a^ ^]^ Only 17 genes were altered by more than twofold in LKO livers, including one of the top upregulated genes, fibroblast growth factor 21 (*Fgf21*) (**Figure** **7**A). The upregulation of *Fgf21* was further confirmed by qPCR (Figure 7B). *Fgf21* encodes a hormone that plays a key role in regulating energy homeostasis.^[^ ^17^ ^]^ Moreover, FGF21 has been shown to enhance insulin sensitivity by increasing glucose uptake in BAT and WAT.^[^ ^18^ ^]^ Therefore, loss of *Lpcat3* in the liver likely improves systemic insulin sensitivity and metabolism via the effects of circulating FGF21, namely, enhanced energy expenditure and glucose uptake in adipose tissues. Consistent with increased *Fgf21* expression in the liver, serum FGF21 levels trended toward an increase in chow diet‐fed LKO mice (Figure 7C). Similarly, hepatic *Fgf21* mRNA and serum FGF21 levels were increased by approximately threefold and more than sixfold in HFD‐fed LKO mice compared to control mice, respectively (Figure 7D,E). The activation of FGF21 signaling was supported by the phosphorylation of its downstream extracellular signal‐regulated kinase (ERK) in WAT of LKO mice (Figure 7F). Next, we measured energy metabolism in HFD‐fed mice by indirect calorimetry using the Comprehensive Lab Animal Monitoring System (CLAMS). LKO mice showed a dramatic increase in energy expenditure (Figure 7G,H), while physical activity and respiratory exchange ratio (RER) were not affected (Figure 7I,J). Taken together, these results demonstrate that loss of *Lpcat3* protects mice from diet‐induced obesity likely through increased energy expenditure.

Similarly, *Fgf21* expression was upregulated in the livers of OLKO mice (Figure S5A, Supporting Information). Serum FGF21 levels were increased by ≈1.6‐fold in OLKO mice compared to OB mice (Figure S5B, Supporting Information). However, we did not observe any difference between OB and OLKO mice in energy expenditure, physical activity and RER in CLAMS analysis (Figure S5C–F, Supporting Information), likely because *ob/ob* mice have much higher basal FGF21 levels and are FGF21 resistant.^[^ ^19^ ^]^

### 2.8 FGF21 Mediates the Effects of Lpcat3 Deficiency on Energy Expenditure, but not Insulin Sensitivity

**Figure 8:**
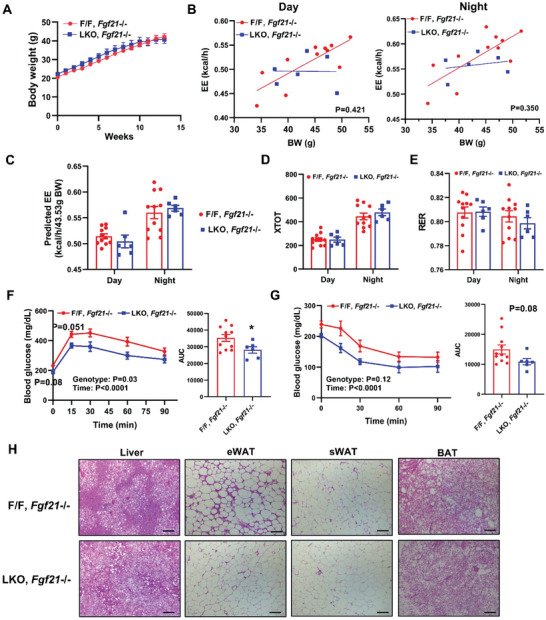
FGF21 mediates the effects of *Lpcat3* deficiency on energy expenditure (EE) but not insulin sensitivity. A) Growth curve of *Lpcat3^fl/fl^,Cre‐, Fgf21* ^−/−^ (F/F, *Fgf21* ^−/−^) and *Lpcat3^fl/fl^, Cre+, Fgf21* ^−/−^ (LKO, *Fgf21* ^−/−^) mice on HFD (*n* = 6 and 11/group). B) EE analyzed by regression analysis of covariance (ANCOVA). C) EE estimated by univariate generalized linear model (GLM) with body mass set to 43.53 g (average body mass of F/F, *Fgf21* ^−/−^ and LKO, *Fgf21* ^−/−^ mice on HFD). D) Physical activity and E) respiratory exchange ratio (RER) in F/F, *Fgf21* ^−/−^ and LKO, *Fgf21* ^−/−^ mice fed with HFD. F,G) GTT and ITT analyses of HFD fed F/F, *Fgf21* ^−/−^ and LKO, *Fgf21* ^−/−^ mice. H) Representative histology of liver, epididymal white adipose tissue (eWAT), subcutaneous white adipose tissue (sWAT) and BAT from HFD fed F/F, *Fgf21* ^−/−^ and LKO, *Fgf21* ^−/−^ mice. Scale bar: 100 µm. Data are presented as means ± SEM. Statistical analysis was performed with Student's *t*‐test (A, AUC in G and H), B) ANCOVA, and C–G) two‐way ANOVA. *P < 0.05.

To determine if loss of *Lpcat3* increases energy expenditure and systemic insulin sensitivity through FGF21, we crossed *Fgf21* ^−/−^ mice with LKO mice to generate *Lpcat3^fl/fl^*, *Cre‐*, *Fgf21* ^−/−^ (F/F, *Fgf21* ^−/−^) and *Lpcat3^fl/fl^*, *Cre+*, *Fgf21* ^−/−^ (LKO, *Fgf21* ^−/−^) mice. *Fgf21* deletion blunted the protection of LKO mice from HFD‐induced obesity (**Figure** **8**A). Consistently, CLAMS analysis showed that enhanced energy expenditure in HFD‐fed LKO mice were abolished by *Fgf21* depletion (Figure 8B,C), whereas physical activity and RER remained unaltered (Figure 8D,E). Interestingly, GTT and ITT analyses demonstrated that loss of *Fgf21* had little impact on the improved insulin sensitivity in HFD‐fed LKO mice. In fact, compared to the controls, LKO, *Fgf21* ^−/−^ mice remained insulin sensitive (Figure 8F,G). Western blot analysis revealed that p‐AKT levels were significantly higher in the livers and WAT of LKO, *Fgf21* ^−/−^ mice compared to those in F/F, *Fgf21* ^−/−^ mice (Figure S6, Supporting Information). Histology analysis revealed that LKO, *Fgf21* ^−/−^ mice had less infiltration of inflammatory cells in WAT compared to F/F, *Fgf21* ^−/−^ mice (Figure 8H), which may contribute to the enhanced insulin sensitivity in LKO independent of FGF21. These data indicate that FGF21 mediates the effect of *Lpcat3* deficiency on energy homeostasis, but not the enhanced insulin sensitivity in LKO mice.

## 3 Discussion

A central paradox of type 2 diabetes is selective insulin resistance, in which insulin fails to suppress hepatic glucose production but continues to stimulate lipogenesis, resulting in hyperglycemia and hypertriglyceridemia.^[^ ^5^ ^]^ However, the underlying mechanisms are unclear. Here we showed that membrane PL composition, controlled by LPCAT3, modulates insulin action on both glucose and lipid metabolism in the liver, and that upregulation of *Lpcat3* expression by hyperinsulinemia and the increase in PL unsaturation are involved in the pathogenesis of insulin resistance, especially hepatic selective insulin resistance. Deletion of *Lpcat3* ameliorates insulin resistance and blunts lipogenesis in both diet‐ and obesity‐induced models, thus alleviating the deleterious effects of insulin resistance on both glucose and lipid metabolism. We further showed that membrane PLs regulate insulin signaling by directly affecting INSR trafficking, and indirectly modulating systemic insulin sensitivity through the action of FGF21 on peripheral tissues. These findings highlight a novel regulatory mechanism of insulin sensitivity by membrane PL composition at both cellular and systemic levels.

Obesity and nonalcoholic fatty liver disease (NAFLD) are major risk factors for insulin resistance and T2D. Hepatic lipid accumulation is crucial for the development of hepatic insulin resistance.^[^ ^11^ ^]^ The levels of several lipids, including FFAs, DAG, ceramides, and triglycerides, inversely correlate with insulin sensitivity. Studies have demonstrated that saturated, but not unsaturated, FAs induce insulin resistance likely through stimulating inflammation and increasing ceramide biosynthesis in muscle and adipose tissue.^[^ ^20^ ^]^ Unlike FFAs, whose saturation impairs insulin sensitivity, our data suggest that increasing membrane PL saturation improves insulin sensitivity and protects mice from diet induced obesity, challenging the conventional notion that saturated fat is always harmful for the body. Furthermore, although LKO mice had higher ceramide, DAG and triglyceride accumulation in liver, they exhibited reduced HGP during insulin infusion and were more insulin sensitive. These data strongly suggest that loss of *Lpcat3* in the liver is sufficient to overcome the detrimental effect of lipid accumulation on insulin signaling, and that hepatic membrane PL composition could be a more prominent determinant of insulin sensitivity. It has been recognized that some patients with NAFLD do not develop obesity and insulin resistance, even though they have higher lipid content in the liver.^[^ ^21^ ^]^ Notably, several recent studies have shown that some of these patients have reduced polyunsaturated PLs in the liver, similar to the lipid profile of LKO livers.^[^ ^22^ ^]^ Thus, our study shed light on the underlying mechanisms of how these patients maintain insulin sensitivity despite massive lipid accumulation.

Our observation that insulin stimulates *Lpcat3* expression and membrane PL composition modulates insulin sensitivity suggest a reciprocal regulation between insulin and membrane PL remodeling that may have physiological and pathological functions. Under physiological conditions, such as fasting/refeeding, insulin increases *Lpcat3* expression, and membrane polyunsaturated PLs, which promote lipogenesis and the conversion of carbohydrates into fatty acids. Under insulin resistance, hyperinsulinemia leads to an excessive increase in *Lpcat3* expression and membrane unsaturation, which enhances lipogenesis and VLDL secretion, resulting in hypertriglyceridemia. On the other hand, increased membrane unsaturation, in turn, blunts insulin signal transduction, thus providing negative feedback to attenuate insulin signaling. The aberrant negative feedback likely leads to insufficient suppression of HGP and hyperglycemia.

Signal transduction has been shown to be influenced by lipid organization of subdomains surrounding receptors.^[^ ^23^ ^]^ There are contradictory reports regarding the localization of INSR in plasma membrane. Some studies claim that INSR is localized in more saturated, detergent resistant raft domains,^[^ ^24^ ^]^ while others show that it is primarily in more fluid, nonraft regions and will translocate to raft domains upon insulin binding.^[^ ^25^ ^]^ Our data showed that INSR is mainly present in the nonraft fraction. We did not observe noticeable translocation of INSR after insulin stimulation. Therefore, the organization of lipid raft in plasma membrane is unlikely involved in the activation of INSR in LKO livers. It is well documented that early endosomes are essential sites of signal transduction for receptor kinase mediated signaling pathways, including INSR.^[^ ^15^, ^26^ ^]^ Our data support the notion that changes in biophysical properties influence the endocytosis of INSR, thereby facilitating signal transduction. However, detailing these biophysical changes and elucidating how they influence INSR movement will require further study with specialized tools and systems. Nevertheless, our data suggest that membrane dynamics may play an important role in insulin signaling in vivo.

Another interesting finding of this study was the profound induction of FGF21 in LKO mice, which is a critical mediator of the systemic effects of hepatic *Lpcat3* deficiency. FGF21 is a hormone with pivotal functions in the regulation of energy homeostasis and metabolism.^[^ ^17^ ^]^ Chronic FGF21 treatment in diet‐induced obese mice promotes thermogenesis and energy expenditure, resulting in weight loss.^[^ ^27^ ^]^ Our data demonstrated that the reduced obesity in LKO mice could be attributed to increased energy expenditure. It has been demonstrated that FGF21 improves insulin sensitivity by increasing glucose uptake in BAT and WAT.^[^ ^18^ ^]^ Surprisingly, LKO mice remain insulin sensitive in the absence of FGF21, indicating that *Lpcat3* deficiency enhances insulin signaling in the liver and adipose tissue independent of FGF21.

In conclusion, our study unveils LPCAT3 as a novel mediator of reciprocal regulation between phospholipid remodeling and insulin signaling that contributes to the pathogenesis of insulin resistance. These findings support an emerging view of dynamic manipulation of membrane biophysical properties as a regulatory mechanism in the control of metabolism.

## 4 Experimental Section

### Mice

All animal procedures were conducted in compliance with protocols approved by the Institutional Animal Care and Use Committee at University of Illinois at Urbana‐Champaign (Protocol #21200). *Lpcat3^fl/fl^* and *Lpcat3^fl/fl^*; *Albumin‐Cre* mice have been described before.^[^ ^10a^ ^]^ *ob/ob* and *Fgf21* ^−/−^ mice were acquired from the Jackson Laboratory. All mice were housed under pathogen‐free conditions in a temperature‐controlled room with a 12 h light/dark cycle. Mice were fed chow diet (LabDiet #5001) or high‐fat diet (Research Diets #D12492). All experiments were performed with male mice unless otherwise stated. For RNA and whole liver protein analysis, liver tissues were collected and snap frozen in liquid nitrogen and stored at −80 °C. In the fasting and refeeding experiments, the fasting group was fasted overnight, and the refeeding group was fasted overnight and then refed with an HFD for 2 h. For food intake measurement, mice were housed individually in cages and food weight was measured daily. Mouse blood was collected by retro‐orbital bleeding before sacrificing or by tail bleeding, and plasma was obtained by centrifugation. Plasma insulin and FGF21 levels were measured using Insulin enzyme‐linked immunosorbent assay (ELISA) kit (Crystal Chem) and Mouse/Rat FGF21 ELISA kit (BioVendor), respectively. For in vivo insulin signaling, insulin (Novolin N, Human) was injected to anesthetized mice retro‐orbitally, and the liver was then perfused with phosphate‐buffered saline (PBS) from *vena cava* and harvested at different time points.

### Glucose and Insulin Tolerance Test

Mice were fasted for at least 5 h in the morning and intraperitoneally (i.p.) injected with glucose or insulin (Novolin N, Human). Glucose was given at 1 g kg^−1^ BW diluted in PBS. Insulin was given at 0.5 U kg^−1^ BW for chow diet‐fed mice and 1 U kg^−1^ BW for HFD‐fed and *ob/ob* mice. Blood glucose levels were measured from tail bleeding using a glucometer (OneTouch Ultra2) at designated time points.

### Indirect Calorimetry and Body Composition Measurements

Metabolic rates were measured by indirect calorimetry in open‐circuit Oxymax chambers in the CLAMS (Columbus Instruments). Mice were housed individually in the chamber, and O_2_ gas was calibrated before monitoring. The chamber was maintained at 23 °C with 12 h light/12 h dark cycles, and food and water were available ad libitum. O_2_ consumption and CO_2_ production were measured directly as continuously accumulated data. EE was calculated as (3.815 + 1.232*RER)*VO_2_ and linear regression analysis of covariance (ANCOVA) was used to determine mass‐independent effect between F/F and LKO mice as reported before.^[^ ^28^ ^]^ Estimated EE was calculated by univariate generalized linear model (GLM) with body mass set to the average of both control and LKO mice. Body composition (whole‐body fat and lean mass) was determined using Echo MRI Body Composition Analyzer.

### Phospholipid Analyses

Mouse livers were snap‐frozen in liquid nitrogen. Livers were homogenized on ice in phosphate buffered saline. Homogenates were subsequently subjected to a modified Bligh‐Dyer lipid extraction^[^ ^29^ ^]^ in the presence of lipid class internal standards, including 1‐0‐heptadecanoyl‐sn‐glycero‐3‐phosphocholine, 1,2‐dieicosanoyl‐sn‐glycero‐3‐phosphocholine.^[^ ^30^ ^]^ Lipid extracts were diluted in methanol/chloroform (4/1, v/v), and molecular species were quantified using electrospray ionization mass spectrometry on a triple quadrupole instrument (Themo Fisher Quantum Ultra) employing shotgun lipidomic methodologies.^[^ ^31^ ^]^ PC molecular species were quantified as chlorinated adducts in the negative ion mode using neutral loss scanning for 50 amu (collision energy = 24 eV). Individual molecular species were quantified by comparing the ion intensities of the individual molecular species to that of the lipid class internal standard with additional corrections for type I and type II 13C isotope effects.^[^ ^31^ ^]^ Each molecular species indicated in Figure 1C was verified by concomitant analyses of samples using product ion scanning for individual fatty acid constituents, including palmitoleic, palmitate, linoleate, oleate, stearate, arachidonate, and docosohexadecanoate (product ion scans in *m*/*z* of 253.2, 255.2, 279.3, 281.3, 283.3, 303.4, and 327.4, respectively, at a collision energy + 35 eV).

### Endosome Isolation

Liver endosomes were isolated as described.^[^ ^32^ ^]^ Briefly, liver tissues were homogenized in homogenization buffer (HB) (250 mm sucrose, 3 mm imidazole pH = 7.4, 1 mm ethylenediaminetetraacetic Acid (EDTA), supplemented with protease and phosphatase inhibitors) using Dounce tissue grinder. The homogenate was centrifuged at 1600 g for 10 min at 4 °C to remove cell debris. Then, the supernatant was mixed thoroughly with 62% sucrose at a ratio of 1:1.36 (Homogenate:sucrose, v/v), overlaid with 1.5 volumes of 35% sucrose, 1 volume of 25% sucrose and finally the tube was filled up with HB. The samples were then centrifuged at 21 000 ×*g* for 3 h at 4 °C. Six fractions were collected from the top for western blot analysis.

### INSR Compartmentation Assay

Membrane and cytosol fractionation was performed as previously described.^[^ ^33^ ^]^ Briefly, tissue samples were homogenized in ice‐cold buffer A (20 mm Tris‐HCl, pH 7.4, 1 mm EDTA, 0.25 mm ethylene glycol tetraacetic acid (EGTA), 250 mm sucrose, and freshly added protease and phosphatase inhibitors, Roche Diagnostics). Debris was removed by centrifugation (5 min, 280 g, 4 °C). The lysate was centrifuged (100 000 g, 60 min at 4 °C), and an aliquot of the supernatant was saved as the cytosolic fraction. The pellet was washed once in ice‐cold buffer B (250 mm Tris‐HCl, pH 7.4, 1 mm EDTA, 0.25 mm EGTA, 2% Triton X‐100, and freshly added protease and phosphatase inhibitors). The pellet was resuspended in buffer B by sonication, incubated at 4 °C for 45 min to solubilize membrane proteins and centrifuged at 100 000 g for 60 min at 4 °C. An aliquot of the supernatant was saved as the membrane fraction.

### TIRF Microscopy Imaging

Primary hepatocytes isolated from chow diet‐fed control and LKO mice were seeded on collagen coated coverslips (Neuvitro GG181.5COLLAGEN), followed by treatment with Cy3 labeled insulin (5 nm) or Alexa 568‐Transferrin (10 µg mL^−1^). Single Molecule tracking was performed immediately after treatments in a custom‐built TIRF microscope (IX73, Olympus) equipped with a 100× oil immersion objective (PlanApo, 100×, N.A. 1.49). A 488 nm (for Cy3‐insulin) or a 561 nm (for Alexa568‐Transferrin) wavelength laser was used as the light source. Power of the laser was controlled using neutral density filters. Individual labeled molecules were seen as diffraction‐limited spots on the cell surface in immediate contact with the coverslip on the glass bottom dish. The fluorescence from the single spots was collected by the same objective, passing an emitter and captured by an electron multiplying charge coupled device camera (iXon U797, Andor Technology). A total of 2000 frames/trajectory was acquired for each field of view with an integration time of 50 ms. Particles in time‐stamped images were analyzed by FIJI.

### Liposome Treatment

All the phospholipids used in the study were purchased from Avanti Polar Lipids. Liposomes were generated as described^[^ ^34^ ^]^ and were composed of DPPC(16:0/16:0)/PAPC(16:0/20:4), 1,2‐distearoyl‐sn‐glycero‐3‐phosphoethanolamine‐N‐[amino(polyethylene glycol) (DSPE‐PEG), and 1,2‐dioleoyl‐3‐trimethylammonium‐propane (DOTAP) at the molar ratio of 76.2:3.8:20. The amount of phospholipid is determined such that the final liposome concentration is 1 mm in PBS whose volume is enough to treat cells. Phospholipids dissolved in chloroform were evaporated under a stream of nitrogen gas and thoroughly dried by rotary vacuum dryer. Dry phospholipids were then hydrated in PBS at a temperature above its transition temperature and vortexed vigorously. The phospholipid solution was passed through 50–80 nm polycarbonate (PCTE) membrane filters (Sterlitech) ten times to make small unilaminar liposomes. Liposomes with different phospholipid composition were treated to primary hepatocytes at final concentration of 100 µm for 15 min before insulin treatment and TIRF imaging.

### Hyperinsulinemic‐Euglycemic Clamp to Assess Insulin Sensitivity in Awake Mice

Hyperinsulinemic–euglycemic clamp experiments were conducted at the National Mouse Metabolic Phenotyping Center (MMPC) at UMass Medical School as previously described.^[^ ^35^ ^]^ In brief, survival surgery was performed 5–6 d before the clamp experiments to establish an indwelling catheter in the jugular vein. On the day of the clamp experiments, mice were fasted overnight (about 17 h), and a 2 h hyperinsulinemic–euglycemic clamp was conducted in awake mice with a primed and continuous infusion of human insulin (150 mU kg^−1^ body weight priming followed by 2.5 mU kg^−1^ min^−1^; Humulin, Eli Lilly). To maintain euglycemia, 20% glucose was infused at variable rates during clamps. Whole‐body glucose turnover and hepatic glucose production were assessed with a continuous infusion of [3–^3^H] glucose (PerkinElmer) before and during the clamp, and 2‐deoxy‐D‐[1‐^14^C] glucose (2‐[^14^C] DG) (PerkinElmer) was administered as a bolus (10 µCi) at 75 min after the start of clamps to measure insulin‐stimulated glucose uptake in individual organs. At the end of the clamps, mice were anesthetized and tissues were taken for biochemical analysis.

Glucose concentrations were analyzed using 5–10 µL plasma and Analox GM9 Analyzer (Analox Instruments). Plasma concentrations of [3–^3^H] glucose, 2‐[^14^C] DG, and ^3^H_2_O were determined following deproteinization of plasma samples as previously described (Kim, 2009). For the determination of 2‐[^14^C] DG‐6‐phosphate content in tissues, tissue samples were homogenized and the supernatants were subjected to an ion‐exchange column to separate 2‐[^14^C] DG‐6‐phosphate from 2‐[^14^C] DG.

Rates of HGP and whole‐body glucose turnover were determined as previously described.^[^ ^35^ ^]^ The insulin‐stimulated rate of HGP was determined by subtracting the glucose infusion rate from the whole‐body glucose turnover. Insulin‐stimulated glucose uptake in individual tissues, skeletal muscle, WAT and BAT, was assessed by determining the content of 2‐[^14^C] DG‐6‐phosphate in each tissue as well as the 2‐[^14^C] DG profile in plasma.

### Statistical Analysis

For all studies, results from quantitative experiments were expressed as means ± standard error of mean (SEM). GraphPad Prism 9.0 (San Diego, CA) was used for all statistical analyses. Where appropriate, significance was calculated by Student's *t*‐test, one‐ or two‐way analysis of variance (ANOVA) with Tukey's or Sidak's multiple comparison test.

## Conflict of Interest

The authors declare no conflict of interest.

## Author Contributions

Y.T. designed and performed experiments, analyzed data, and wrote the paper. K.M., M.J.J., H.S., W.L., R.S., and K.I. performed experiments and analyzed data. R.H.F and J.K.Ki. designed, performed, and analyzed data for clamp study. J.K.Ke., D.A.F., and K.Z. designed experiments and analyzed data. B.W. conceived the project, designed experiments, analyzed data, supervised the project, and wrote the paper.

## Supporting information

Supporting Information

Supplemental Video 1

Supplemental Video 2

Supplemental Video 3

Supplemental Video 4

Supplemental Video 5

Supplemental Video 6

Supplemental Video 7

Supplemental Video 8

## Data Availability

The data that support the findings of this study are available from the corresponding author upon reasonable request.
