# Supplementary material for: Hepatic Phospholipid Remodeling Modulates Insulin Sensitivity and Systemic Metabolism
Source: Adv Sci (Weinh). 2023 Apr 23;10(18):2300416. doi: 10.1002/advs.202300416 (PMC10288282; doi:10.1002/advs.202300416)
Supplement: Supplementary file 1 — Supporting Information [file ADVS-10-2300416-s002.pdf]

Hepatic Phospholipid Remodeling Modulates Insulin Sensitivity and Systemic Metabolism

Ye Tian[1], Kritika Mehta[2], Matthew J. Jellinek[6], Hao Sun[3], Wei Lu[1], Ruicheng Shi[1], Kevin Ingram[2], Randall H. Friedline[7], Jason K. Kim[7], Jongsook Kim Kemper[3,5], David A. Ford[6], Kai Zhang[2], Bo Wang[1,4, 5*]

Supplemental information

This file contains 6 supplemental figures, supplemental materials and methods, supplemental table 1, and captions for supplemental videos.

Figure S1. Hepatic *Lpcat3* deficiency improves HFD-induced insulin resistance.

Figure S2. *Lpcat3* deficiency in the liver improves insulin resistance in *ob/ob* mice.

Figure S3. Shotgun lipidomic analysis of lipids in LKO mouse livers.

Figure S4. Loss of *Lpcat3* does not affect INSR protein levels or its localization.

Figure S5. Loss of *Lpcat3* has no effect on energy expenditure in *ob/ob* mice.

Figure S6. Loss of *Lpcat3* enhances insulin signaling in the liver and adipose tissue independent of FGF21.

Supplemental table 1. List of key reagents and resources.

Video S1. TIRF microscopy analysis of the internalization of Cy3 labeled insulin in LKO hepatocytes.

Video S2. TIRF microscopy analysis of the internalization of Cy3 labeled insulin in control hepatocytes.

Video S3. TIRF microscopy analysis of the internalization of Alexa568-transferrin in LKO hepatocytes.

Video S4. TIRF microscopy analysis of the internalization of Alexa568-transferrin in control hepatocytes.

Video S5. TIRF microscopy analysis of the internalization of Cy3 labeled insulin in control hepatocytes treated with DPPC liposome.

Video S6. TIRF microscopy analysis of the internalization of Cy3 labeled insulin in control hepatocytes treated with PAPC liposome.

Video S7. TIRF microscopy analysis of the internalization of Cy3 labeled insulin in LKO hepatocytes treated with DPPC liposome.

Video S8. TIRF microscopy analysis of the internalization of Cy3 labeled insulin in LKO hepatocytes treated with PAPC liposome.

Supplemental Figures

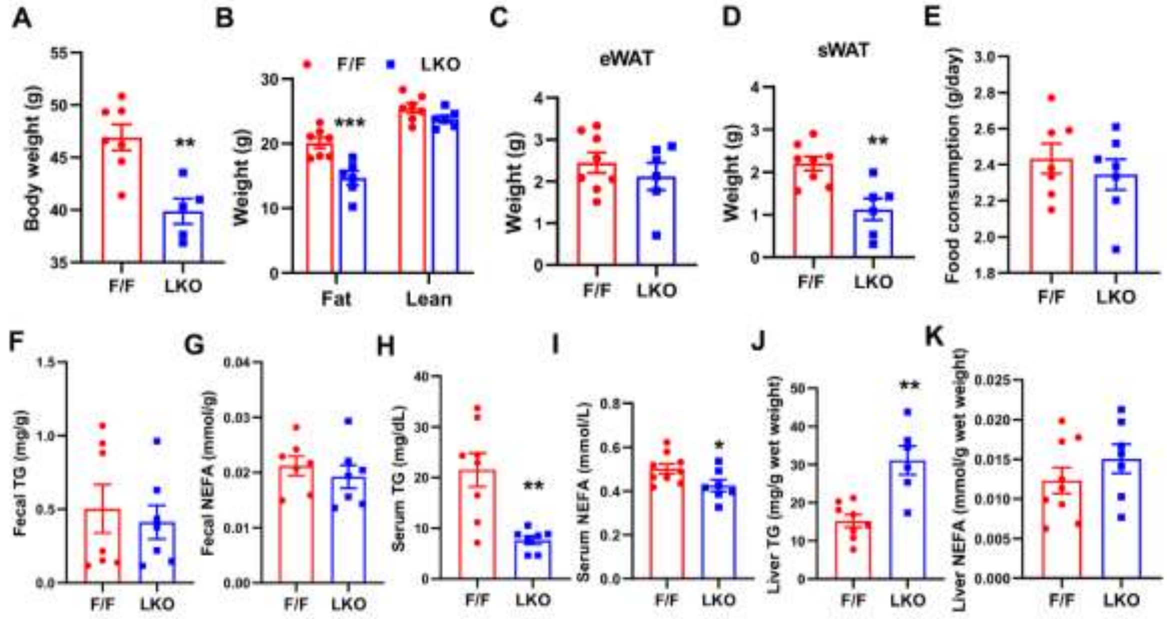

Figure S1. Hepatic *Lpcat3* deficiency improves HFD-induced insulin resistance.

A. End point body weight of HFD-fed F/F and LKO mice.
B-D. Body composition analyzed by Echo-MRI (B) and tissue weight of eWAT (C) and sWAT (D) from HFD-fed F/F and LKO mice.
E. Daily food consumption of F/F and LKO mice on HFD. Data was presented as average of four days.
F-G. Fecal Triglyceride (F) and non-esterified fatty acid (G) levels of F/F and LKO mice on HFD.
H-K. Serum triglyceride (H) and non-esterified fatty acids (NEFA) (I) levels and liver triglyceride (J) and NEFA (K) levels of HFD-fed F/F and LKO mice.
Data are presented as means ± SEM. Statistical analysis was performed with Student's t test (A, C-K) and two-way ANOVA (B). $^{*}P < 0.05$, $^{**}P < 0.01$, $^{***}P < 0.001$.

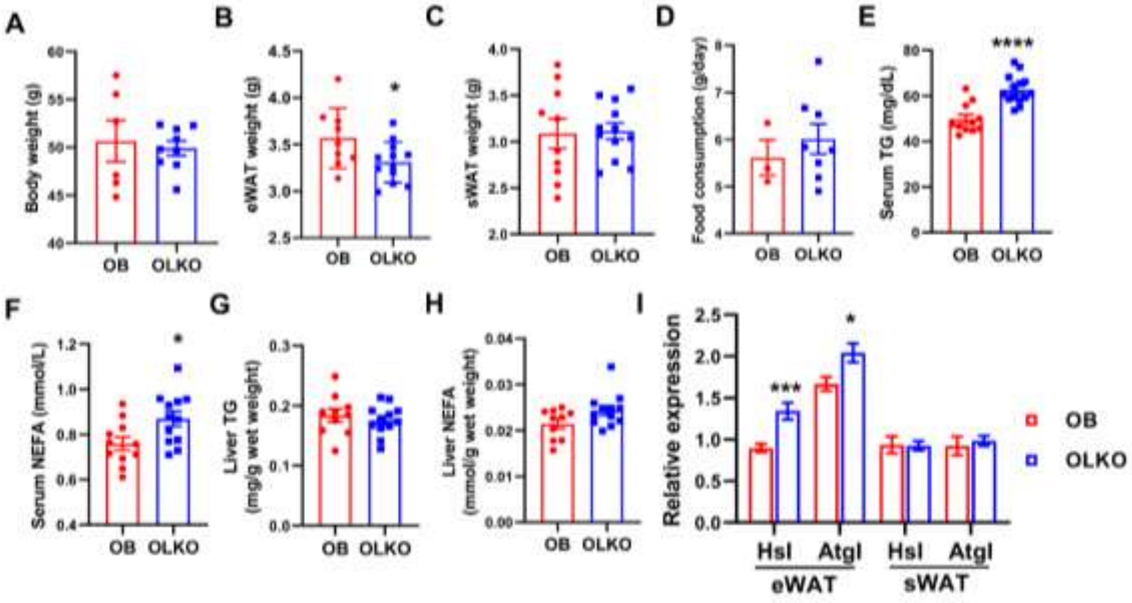

Figure S2. *Lpcat3* deficiency in the liver improves insulin resistance in *ob/ob* mice.

A. End point body weight of *ob/ob Lpcat3*$^{fl/fl}$ (OB) and *ob/ob Lpcat3*$^{fl/fl}$ *Albumin-Cre* (OLKO) mice.

B-C. Tissue weight of eWAT (B) and sWAT (C) in OB and OLKO mice.

D. Daily food consumption of OB and OLKO mice. Data was presented as average of three days.

E-H. Serum triglyceride (E) and non-esterified fatty acids (NEFA) (F) levels and liver triglyceride (G) and NEFA (H) levels of OB and OLKO mice.

I. Expression of lipases in eWAT and sWAT of OB and OLKO mice (n≥10/group).

Data are presented as means ± SEM. Statistical analysis was performed with Student’s t test. *P < 0.05, ***P < 0.001, ****P < 0.0001.

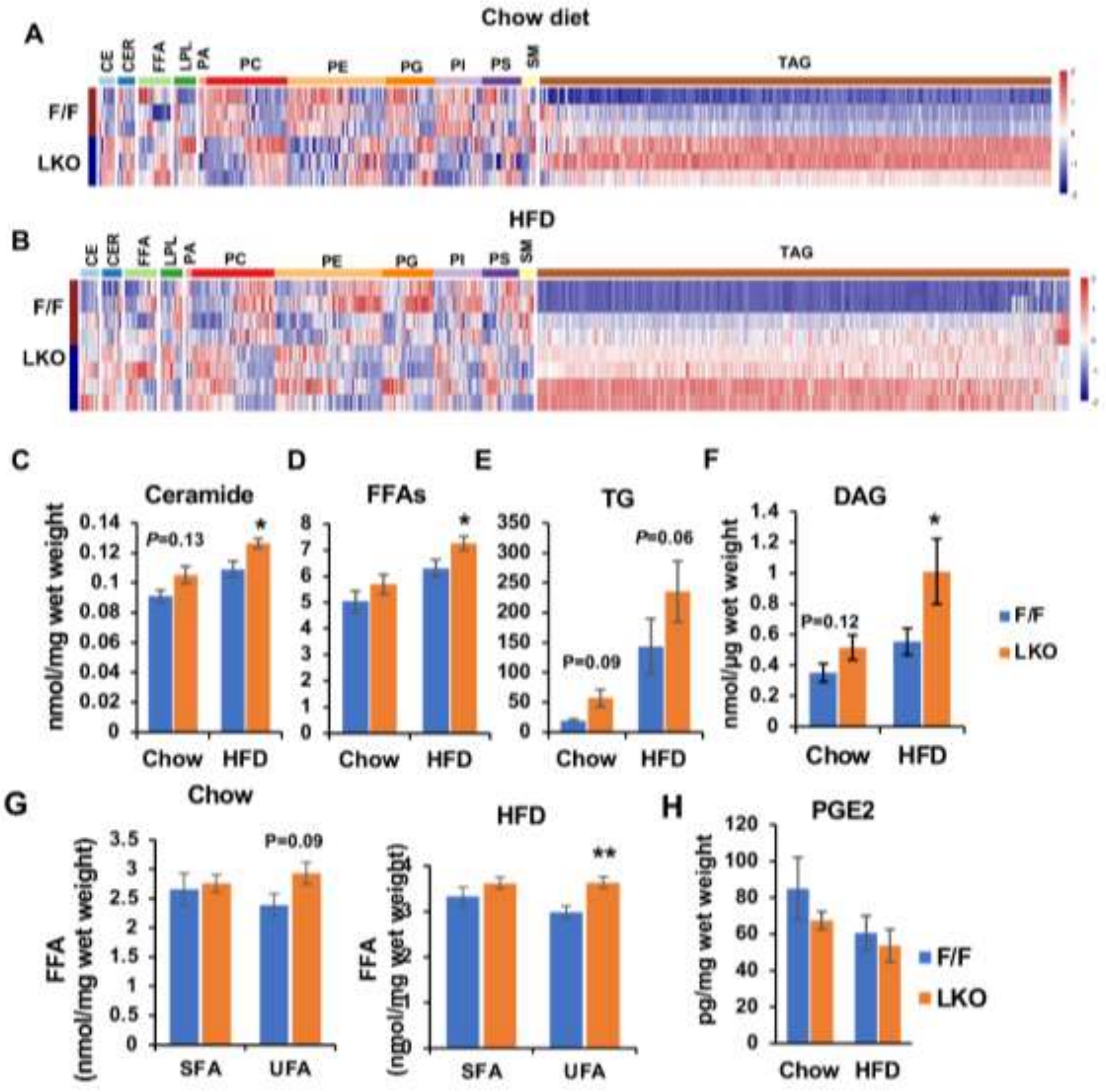

Figure S3. Shotgun lipidomic analysis of lipids in LKO mouse livers.

A-B. Heatmap of lipid classes in the livers of F/F and LKO mice fed chow (A) and HFD (B) (n=3-4/group).
C-E. Lipidomic quantification of ceramides, free fatty acids (FFAs) and triacylglycerides (TAGs) in the livers of F/F and LKO mice fed chow and HFD (n=3-4/group).
F. Quantification of diacylglycerol (DAG) in the livers of F/F and LKO mice fed chow and HFD (n=5-8/group).
G. Lipidomic quantification of saturated FFA (SFA) and unsaturated FFA (UFA) in the livers of F/F and LKO mice fed chow and HFD (n=3-4/group).
H. Liver prostaglandin E2 levels of *Lpcat3*$^{fl/fl}$ (F/F) and *Lpcat3*$^{fl/fl}$ *Albumin-Cre* (LKO) mice fed on chow and HFD (n=4-6/group).
Data are presented as means ± SEM. Statistical analysis was performed with Student's t test. *P < 0.05, ** P < 0.05.

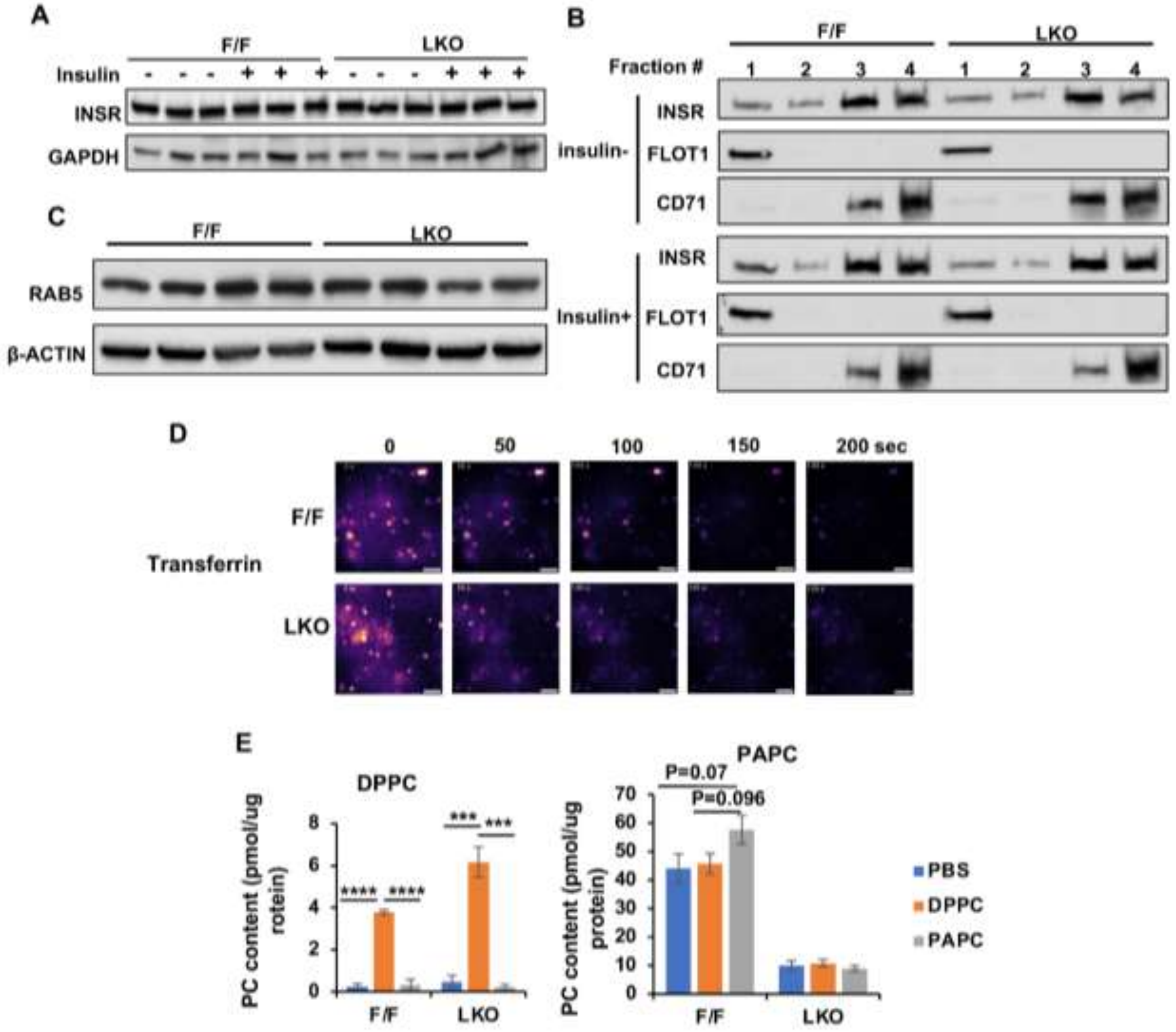

Figure S4. **Loss of *Lpcat3* does not affect INSR protein levels or its localization.**

A. Western blot analysis of total insulin receptor (INSR) levels in female chow diet-fed F/F and LKO mouse livers.
B. Western blot analysis of INSR localization in different microdomains of hepatic plasma membrane before and after insulin treatment. Detergent resistant and non-resistant fractions were purified from chow diet-fed F/F and LKO mice before and after retro-orbital injection of insulin (1 U/kg BW).
C. Western blot analysis of RAB5 levels in whole tissue lysates from F/F and LKO mouse livers.
D. Representative images of TIRF microscopy of primary hepatocytes isolated from F/F and LKO mice and treated with Alexa 568-transferrin.
E. Mass spectrometry analysis of PC content in primary hepatocytes treated with liposomes containing DPPC or PAPC.
Data are presented as means ± SEM. Statistical analysis was performed with one-way ANOVA (E). ***$P < 0.001$, ****$P < 0.0001$.

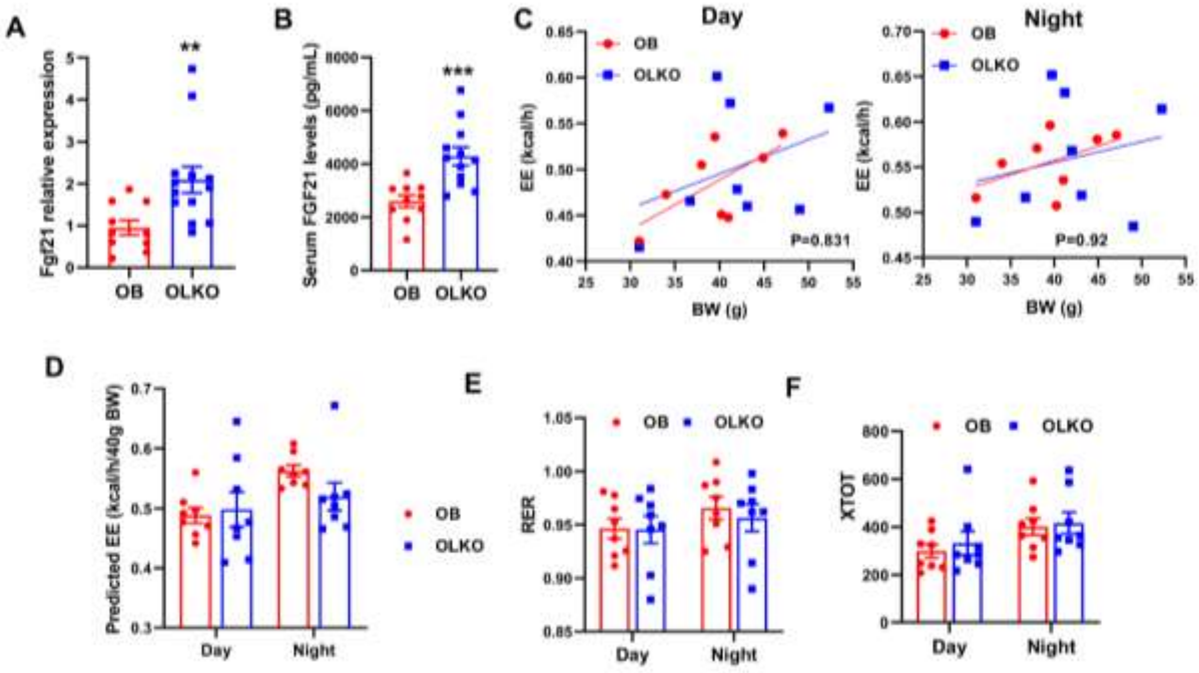

Figure S5. Loss of *Lpcat3* has no effect on energy expenditure in *ob/ob* mice.

A-B. *Fgf21* mRNA levels in the livers (A) and serum FGF21 levels (B) of OB and OLKO mice.
C. Energy expenditure (EE) analyzed by regression analysis of covariance (ANCOVA).
D. EE estimated by univariate generalized linear model (GLM) with body mass set to 40.3 g (average body mass of OB and OLKO mice).
E-F. RER (E) and physical activity (F) of OB and OLKO mice.
Data are presented as means ± SEM. Statistical analysis was performed with Student's t test (A-B), ANCOVA (C) and two-way ANOVA (D-F). $*P < 0.05$, $**P < 0.01$, $***P < 0.001$.

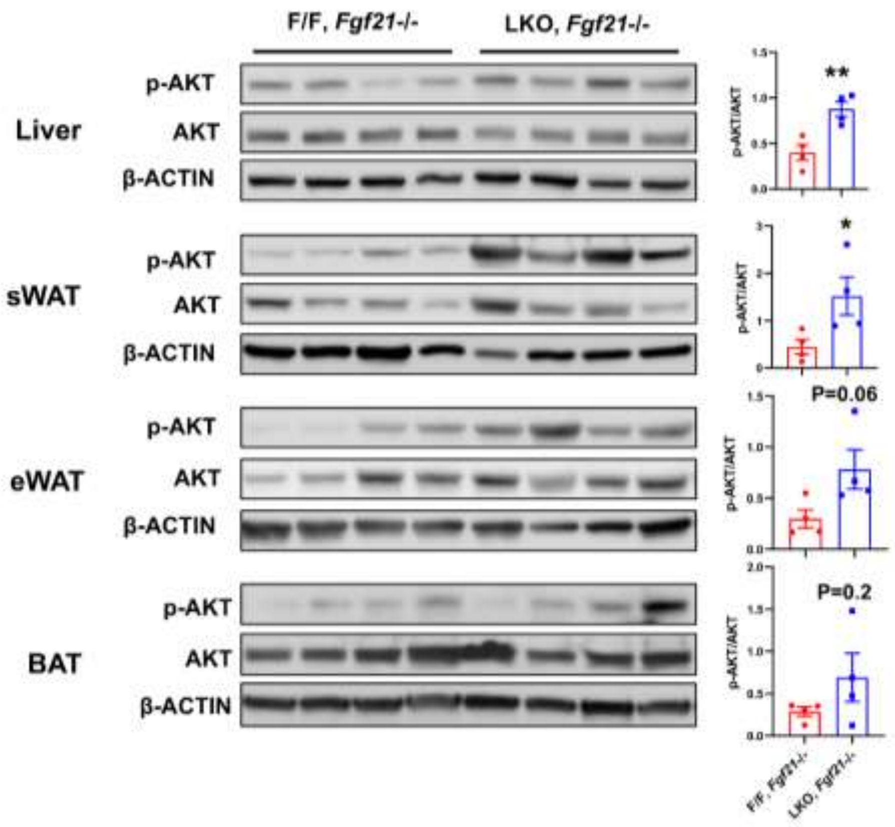

Figure S6. Loss of *Lpcat3* enhances insulin signaling in the liver and adipose tissue independent of FGF21.

Western blot analysis of pAKT (Ser473) levels in HFD-fed F/F, *Fgf21*-/- and LKO, *Fgf21*-/- mouse tissues. Mice were fed HFD for 12 weeks, fasted overnight and i.v. injected with insulin (1 U/kg BW) for 15 min.
Data are presented as means ± SEM. Statistical analysis was performed with Student's t test. *P < 0.05, **P < 0.01.

Supplemental materials and methods

Cell culture

Hepa 1-6 cell line was maintained in Dulbecco's modified Eagle's medium (DMEM) supplemented with 10% fetal bovine serum (FBS), 4.5 g/L glucose, 1 mM sodium pyruvate and 5mM L-glutamine at 37°C with 5% CO_2. Hepa 1-6 cells were treated with insulin (5 nM) for 0, 1, 4 and 12 h, and cells were harvested for gene expression analysis. Primary hepatocytes were isolated as previously described [1]. Briefly, we cannulated the inferior *vena cava* of anesthetized mice and perfused the liver with 37°C liver perfusion medium (0.5 mM EGTA and 10 mM HEPES in Hanks solution) for 5 min, then with 40 µg/mL 37°C Liberase TM in 10 mM HEPES-buffered William's medium E for another 10 min. Perfused and digested liver was removed and placed in William's medium E, and cells were dispersed. We filtered the resulting cell suspension through a 100-µm cell strainer and washed 3 times with William's medium E. Live cells were counted using trypan blue staining. Primary hepatocytes were used only when the viability was above 85%. 1×10^6 live cells were seeded per well of 6-well collagen I–coated plates in William E medium supplemented with 2 mM glutamine, 10 mM HEPES, 1% penicillin and streptomycin (P/S) and 5% FBS. After 3 to 4 hours, cells were washed twice with 2 mL PBS and cultured in maintenance medium (William E medium supplemented with 2mM glutamine, 10 mM HEPES, 1% P/S and 0.2% fatty acid-free BSA).

Histology

Tissues were collected, fixed in 10% buffered formalin, embedded in paraffin, sectioned at 4-10 µm and stained with hematoxylin and eosin at the University of Illinois Histology Lab.

Gene expression analysis

Total RNA was isolated from tissues with Trizol (Invitrogen), cDNA was synthesized, and gene expression was quantified by BioRad CFX384 Touch Real-Time PCR Detection System with SYBR Green (BioRad). For ASO injected mice, total RNA was isolated with Direct-zol RNA Kits (Zymo Research), and cDNA was synthesized using iScript™ cDNA Synthesis Kit (BioRad). The sequences of primers used in this study were listed in supplemental table.

Western-Blotting

Whole cell and liver tissue were homogenized and sonicated in RIPA buffer (50 mM Tris–HCl, pH 7.4, 150 mM NaCl, 0.1% Triton X-100, 0.5% sodium deoxycholate, 0.1% SDS) supplemented with protease inhibitors (Roche Molecular Biochemicals) and phosphatase inhibitors (Thermo Fisher Scientific). Adipose tissue protein extraction was conducted as described previously (An and Scherer, 2020). The homogenate was then cleared by centrifugation. After determining protein concentrations using BCA kits (Thermo Fisher Scientific), 45 µg of proteins were mixed with sample buffer (BioRad), loaded to 4-15% precast gel (BioRad) for electrophoresis and transferred to PVDF membranes (Amersham International, GE Healthcare). Membranes were then blocked with blocking buffer (5% milk or BSA in 0.1% TBST) for 1 h at room temperature and then incubated with primary antibodies (**Supplemental table 1**) diluted in blocking buffer at 4°C overnight. After washing with 0.1% TBST 4 times, membranes were incubated with secondary antibodies diluted in blocking buffer for 1 h at room temperature. Membranes were developed using ECL western blotting substrate (Thermo Scientific) and were exposed to X-ray film (FUJI) in a dark room. Antibodies used in this study were listed in supplemental table.

Detergent resistant membrane (DRM) isolation

Mouse liver DRM was isolated as previously described [2], with modifications. Briefly, 50-100 mg fresh livers were collected and homogenized on ice with Dounce tissue grinder in TNE buffer (150 mM NaCl, 2 mM EDTA, 50 mM Tris–HCl, pH 7.4) supplemented with protease and phosphatase inhibitors. The homogenate was centrifuged at 380 g for 5 min at 4°C to get rid of tissue debris and intact cells. The supernatant was mixed with 2X volume of OptiPrep (60% iodixanol), overlaid with 30% iodixanol, then with TNE in a TLS55 centrifuge tube and centrifuged at 55,000 r.p.m. for 2 h. The fraction in the upper interface was collected as the DRM sample.

Lipid extraction and measurement

Liver tissues were snap-frozen in liquid nitrogen at the time of harvest. To extract lipids, liver tissues were cut, weighed and homogenized in water. After transferring the homogenate into glass tubes, lipids were extracted by adding 2 mL chloroform/methanol (2:1 v/v), mixed thoroughly by vortexing and centrifuged at 3,000 r.p.m. for 5 min at 4°C. Lipids in the lower layer were then carefully collected and air-dried overnight. The lipids were dissolved in ethanol and diluted in PBS for lipid measurements. Serum was directly used for lipid measurements. Serum and hepatic lipids were measured with the Wako L-Type TG M kit and the Wako HR series NEFA- HR(2) kit.

Liver DAG measurement

Around 10 mg liver samples were homogenized in PBS. Lipid was then extracted and DAG concentration was measured following manufacturer's instructions. The final DAG concentration was normalized to sample wet weight.

Hepatic glucose production

Primary hepatocytes were isolated and cultured overnight in collagen-coated 12-well plates. Then, change culture media to fasting media (DMEM without glucose, glutamine and phenol red) to fast cells for 6 hours. The glucose production was stimulated by adding 20mM lactate and 2mM pyruvate into the media with or without 10 nM insulin. The media was then collected 3 hours after treatment and the glucose concentration was measured using a high sensitive glucose kit. Hepatic glucose production rate was normalized to protein quantity of each well.

Lipidomic assay

Lipidomic analyses were carried out at the University of California, Los Angeles (UCLA) lipidomics core. The UCLA lipidomics core protocol has been previously described [3]. In brief, for tissue, 50-100 mg of frozen liver were placed in a 2 mL homogenizer tube pre-loaded with 2.8 mm ceramic beads (Omni #19-628). PBS was added to the tube, and the sample was homogenized in the Omni Bead Ruptor Elite (3 cycles of 10 seconds at 5 m/s with a 10 second dwell time). For lipid extraction, 3-6 mg of tissue homogenate were transferred to a glass tube for lipid extraction using a modified Bligh and Dyer extraction [4]. Prior to biphasic extraction, a 13-lipid class Lipidyzer Internal Standard Mix was added to each sample (AB Sciex, 5040156). Following two successive extractions, pooled organic layers were dried down using a Genevac EZ-2 Elite. Lipid samples were resuspended in 1:1 methanol/dichloromethane with 10 mM Ammonium Acetate and transferred to robovials (Thermo 10800107) for analysis. Samples were analyzed on the Sciex Lipidyzer Platform for targeted quantitative measurement of 1100 lipid species across 13 classes. Differential Mobility Device on Lipidyzer was tuned with SelexION tuning kit (Sciex 5040141). Instrument settings, tuning settings and the MRM list available upon request. Data analysis was performed on the Lipidyzer software. Quantitative values were normalized to mg of liver.

Prostaglandin E2 (PGE2) measurement

Mouse livers were harvested and snap-frozen in liquid nitrogen. Frozen samples were homogenized in 500 μL of cold PBS containing indomethacin (10 mg/mL) using a tissue homogenizer. The suspension was sonicated, followed by centrifugation for 10 minutes at 12,000 r.p.m. PGE2 levels in the supernatant were measured using a PGE2 ELISA Kit (Thermo Fisher Scientific).

Supplemental table 1. List of key reagents and resources.

REAGENT or RESOURCE	SOURCE	Catalog #
**Antibodies**		
Rabbit monoclonal anti-phospho-AKT	Cell Signaling	4060S
Rabbit polyclonal anti-AKT	Cell Signaling	9272S
Rabbit monoclonal anti-INSR	Cell Signaling	3025T
Rabbit monoclonal anti- phosphor-INSR	Cell Signaling	3024T
Mouse monoclonal anti-GAPDH	Millipore	MAB374
Rabbit monoclonal anti-β-actin	Cell Signaling	4970S
Rabbit monoclonal anti-RAB5	Cell Signaling	3547T
Rabbit monoclonal anti-phospho-ERK	Cell Signaling	4370S
Rabbit monoclonal anti-ERK	Cell Signaling	4695
Rabbit monoclonal anti-FLOT1	Cell Signaling	18634S
Mouse monoclonal anti-CD71	Santa Cruz Biotechnology	sc-65882
IgG (H+L) Goat anti-Rabbit, HRP	Invitrogen	656120
Goat anti-Mouse IgG(H+L) Secondary Antibody, HRP	Invitrogen	31430
**Chemicals, Peptides, and Recombinant Proteins**		
Insulin, human recombinant, zinc solution (for cell treatments)	Thermo Fisher	12585014
Novolin® human insulin (for mouse experiments)	Novo Nordisk	0169-1833-11
Insulin (Human) - Cy3 Labeled	Phoenix Pharmaceuticals	FC3-035-06
Transferrin From Human Serum, Alexa Fluor™ 568 Conjugate	Invitrogen	T23365
**Critical Commercial Assays**		
Prostaglandin E2 Parameter Assay Kit	R&D Systems	KGE004B
Ultra Sensitive Mouse Insulin ELISA Kit	Crystal Chem	MSPP-90080
Fibroblast Growth Factor 21, FGF21, Mouse/Rat ELISA	BioVendor	102552-226
DAG assay kit	Cell Biolabs, Inc	MET-5028
**Deposited Data**		
Microarray data	Rong et al., 2015	GSE98516
**Experimental Models: Organisms/Strains**		
Mouse: Lpcat3 Floxed Alb-Cre mice	Rong et al., 2015	N/A

Mouse: C57BL/6J	The Jackson Laboratory	000664
Mouse: B6.Cg-*Lep*^{ob}/J	The Jackson Laboratory	000632
Mouse: B6.129Sv(Cg)-Fgf21tm1.1Djm/J	The Jackson Laboratory	033846
**Oligonucleotides (qPCR primers)**		
m36B4-F AGATGCAGCAGATCCGCAT		
m36B4-R GTTCTTGCCCATCAGCACC		
mLpcat3-F GGCCTCTCAATTGCTTATTTCA		
mLpcat3-R AGCACGACACATAGCAAGGA		
mFasn-F ACCACCAGAGACCGTTATGC		
mFasn-R CAGCAGAGTCTACAGCTACCT		
mFgf21-F CACCCAGGATTTGAATGACC		
mFgf21-R CTGGGGGTCTACCAAGCATA		
mElovl6-F ACAATGGACCTGTCAGCAAA		
mElovl6-R GTACCAGTGCAGGAAGATCAGT		
mTnfα-F TGCCTATGTCTCAGCCTCTTC		
mTnfα-R GAGGCCATTTGGGAACTTCT		
mPepck-F CTGCATAACGGTCTGGACTTC		
mPepck-R CAGCAACTGCCCGTACTCC		
mG6pc-F CGACTCGCTATCTCCAAGTGA		
mG6pc-R GTTGAACCAGTCTCCGACCA		
mGys2-F CCAGCTTGACAAGTTCGACA		
mGys2-R ATCAGGCTTCCTCTTCAGCA		
mCcl3-F CAGGCATTCAGTTCCAGGTC		
mCCl3-R TGCCCTTGCTGTTCTTCTCT		
mCd68-F CTTCCCACAGGCAGCACAG		
mCd68-R AATGATGAGAGGCAGCAAGAGG		
mF4/80-F CTTTGGCTATGGGCTTCCAGTC		
mF4/80-R GCAAGGAGGACAGAGTTTATCGTG		
mSrebf1c-F GGAGCCATGGATTGCACATT		
mSrebf1c-R GGCCCGGGAAGTCACTGT		
mScd1-F CGAAGTCCACGCTCGATCTC		
mScd1-R TGTGGGCCGGCATGAT		
mHsl-F CCAGCCTGAGGGCTTACTG		
mHsl-R CTCCATTGACTGTGACATCTCG		
mAtgl-F GGATGGCGGCATTTCAGACA		
mAtgl-R CAAAGGGTTGGGTTGGTTCAG		
mAcaca-F GATGAACCATCTCCGTTGGC		
mAcaca-R GACCCAATTATGAATCGGGAGTG		
**Software and Algorithms**		
GraphPad Prism	GraphPad Software	https://www.graphpad.com/scientificsoftware/prism/
R	R Core Team	https://www.r-project.org/
ImageJ	NIH	https://imagej.nih.gov/ij/

References

[1] X. Rong, B. Wang, E. N. Palladino, T. Q. de Aguiar Vallim, D. A. Ford, P. Tontonoz, *J Clin Invest* **2017**, *127* (10), 3640, https://doi.org/10.1172/JCI93616.

[2] D. Lingwood, K. Simons, *Nat Protoc* **2007**, *2* (9), 2159, https://doi.org/10.1038/nprot.2007.294.

[3] W. Y. Hsieh, K. J. Williams, B. Su, S. J. Bensinger, *STAR Protoc* **2021**, *2* (1), 100235, https://doi.org/10.1016/j.xpro.2020.100235.

[4] E. G. Bligh, W. J. Dyer, *Can J Biochem Physiol* **1959**, *37* (8), 911, https://doi.org/10.1139/o59-099.
